# Multiblock Thermoplastic Polyurethanes: In Situ Studies of Structural and Morphological Evolution under Strain

**DOI:** 10.3390/ma14113009

**Published:** 2021-06-01

**Authors:** Denis V. Anokhin, Marina A. Gorbunova, Ainur F. Abukaev, Dimitri A. Ivanov

**Affiliations:** 1 Institute for Problems of Chemical Physics Russian Academy of Sciences, Semenov Prospect 1, 142432 Chernogolovka, Russia; mflute2008@yandex.ru (M.A.G.); abukaev.af@phystech.edu (A.F.A.); dimitri.ivanov.2014@gmail.com (D.A.I.); 2 Faculty of Fundamental Physical and Chemical Engineering, Lomonosov Moscow State University, 1 Leninskie Gory, 119991 Moscow, Russia; 3 Moscow Institute of Physics and Technology, Institutskiy per. 9, 141700 Dolgoprudny, Russia; 4 Institut de Sciences des Matériaux de Mulhouse-IS2M, CNRS UMR 7361, Jean Starcky, 15, F-68057 Mulhouse, France

**Keywords:** thermoplastic polyurethane ureas, shape memory materials, synchrotron SAXS/WAXS, polymer deformation, lamellar morphology, poly-ε-caprolactone, poly(1,4-butylene adipate)

## Abstract

The structural evolution of multiblock thermoplastic polyurethane ureas based on two polydiols, poly(1,4-butylene adipate (PBA) and poly-ε-caprolactone (PCL), as soft blocks and two diisocyanites, 2,4-toluylene diisocyanate (TDI) and 1,6-hexamethylene diisocyanate (HMDI), as hard blocks is monitored during in situ deformation by small- and wide-angle X-ray scattering. It was shown that the urethane environment determines the crystal structure of the soft block. Consequently, two populations of crystalline domains of polydiols are formed. Aromatic TDI forms rigid domains and imposes constrains on the crystallization of bounded polydiol. During stretching, the TDI–polydiol domains reveal limited elastic deformation without reorganization of the crystalline phase. The constrained lamellae of polydiol form an additional physical network that contributes to the elastic modulus and strength of the material. In contrast, polydiols connected to the linear semi-flexible HMDI have a higher crystallization rate and exhibit a more regular lamellar morphology. During deformation, the HMDI-PBA domains show a typical thermoplastic behavior with plastic flow and necking because of the high degree of crystallinity of PBA at room temperature. Materials with HMDI-PCL bonding exhibit elastic deformation due to the low degree of crystallinity of the PCL block in the isotropic state. At higher strain, hardening of the material is observed due to the stress-induced crystallization of PCL.

## 1. Introduction

Recently, a new class of materials adaptive to external factors has started to rapidly develop. They can be exemplified by thermoplastic elastomers based on semi-crystalline polyurethanes (TPU) and polyurethane-ureas (TPUU). Such materials combining elasticity and strength have been already actively used in medicine for the engineering of tissues, plasters, sutures, implants, as well as in other areas [1,2,3]. TPU and TPUU are linear block copolymers consisting of thermodynamically incompatible soft (SB) and hard (HB) blocks that undergo a microphase separation. The soft segment is formed by polyester or polyether diols such as poly-ε-caprolactone (PCL) [4,5], poly(1,4-butylene adipate) (PBA), and their mixtures [6,7], poly-L-lactide [8], poly(ethylene glycol) [9], etc. The hard blocks are diol-urethane or urea fragments synthesized from aromatic diisocyanates (2,4-toluene diisocyanate), cycloaliphatic (isophoron diisocyanate), and aliphatic (1,6-hexamethylenediisocyanate) compounds and chain extenders (1,4-butanediol, diamine, and 2-aminoethanol).

Apart from chemical composition, the mechanical, thermal, and relaxation properties of TPU and TPUU depend on the thermal history [10,11,12]. Thus, semi-crystalline polyurethanes are able to fix their temporary shape during deformation and further restore the original shape after heating above a switching temperature, i.e., they show the shape memory effect (SME) [13,14]. In a previous publication, it was shown that SME is determined by transition of the TPUU between three morphological states: (1) semi-crystalline lamellar morphology of isotropic film in a permanent shape, (2) fibrillar morphology with extended-chain crystals of the soft block after film stretching to a temporary shape, and (3) phase-separated state of the block copolymer in the amorphous state after shape recovery above the switching temperature [15]. At present, the attention of researchers is directed to studies of thermomechanical properties of adaptive TPUU [16,17,18,19]. However, the relationship between the chemical composition, nanoscale morphology, and thermomechanical characteristics is far from being fully understood.

Synchrotron small- and wide-angle X-ray scattering (SAXS/WAXS) is a powerful technique for analysis of polymer structure. The high brilliance of the X-ray source allows monitoring structural and morphological evolution with adequate time resolution. In particular, a combination of SAXS/WAXS with DSC [20,21,22,23,24,25], FTIR [26], and relaxometry [27] is capable of providing important information about structural evolution under external factors.

Structural investigations of TPUs are focused on a combination of two main processes: phase separation and ordering of HB [22,28]. The size and packing quality depend on the type of diisocyanate, its content, and preparation conditions. Particularly, the growth of HB content and annealing at elevated temperature stimulate an association process with an increase of HB domain size and relaxation of internal stress [21,22].

The most efficient approach combines SAXS/WAXS with uniaxial strain at different temperatures. Classical models of deformation of semi-crystalline polymers and block copolymers consider the continuous increase of a long period during stretching [29]. As a result of the specific chemical structure, polyurethanes possess fundamentally different structural evolution during deformation [30,31,32,33]. Particularly, TPU diffractograms often show a retardation of SAXS peak shift during film deformation, which indicates that the morphology is not related to the sequence of hard and soft domains [34,35]. A detailed study of TPU deformation was performed using in situ SAXS/WAXS experiments during deformation [36,37,38].

In studies of TPU based on polytetrahydrofurane (PTHF1000), methylene diphenyl diisocyanate (MDI), and 1,4-butanediol, Stribeck et al. explained a considerable retardation of the SAXS peak with respect to deformation by “paracrystalline” morphology of the HB. The authors suggested that a considerable fraction of the macroscopic strain must be realized in “poorly arranged entities” that do not contribute to the SAXS maximum because of absence of quasiperiodicity. In contrast, “well-arranged ensembles” are hardly deformed during strain and give a main contribution to the SAXS peak. At high deformation, tie polymer chains start to be pulled out from “well-arranged ensembles”, resulting in a release of stress and shrinking of the long period (sacrifice-and-relief mechanism) [39,40].

It should be mentioned that despite numerous publications on structural evolution of TPUs under strain, only a few of them discuss the crystallization of soft block [41,42]. This can be explained by the low soft block content and small molecular weight of polydiols, which are used in most of the studies. Thus, the impact of soft block crystallinity, distribution of crystalline domains, and the size and kinetics of formation on macroscopic properties has been not well studied yet.

In the present work, the structural evolution of multiblock thermoplastic polyurethane urea based on two polydiols (poly(1,4-butylene adipate diol) and poly-ε-caprolactone diol) and two diisocyanites (diisocyanates (2,4-toluylene diisocyanate and 1,6-hexamethylene diisocyanate) is monitored as a function of temperature and strain during in-situ SAXS/WAXS experiments.

## 2. Materials and Methods

### 2.1. Materials

Poly(1,4-butylene adipate) diol (PBA) (Huakai Resin Co., Ltd., Shandong, China, Mn = 2000 Da) and poly-ε-caprolactone diol (PCL) (Merck KGaA, Darmstadt, Germany, Mn = 2000 Da) were dried in vacuum for 4 h at 80 °C. The OH groups content determined by chemical method [43] was 1.7 w/w%. Diisocyanates (2,4-toluylene diisocyanate (TDI) and 1,6-hexamethylene diisocyanate (HMDI)) (Merck KGaA, Darmstadt, Germany) were distilled in vacuum at 50–55 °C/12 mm Hg and stored in sealed ampoules. Chain elongation agents (2-amino-1-ethanol (EA) and 1,4-butanediol (BD)) were distilled over freshly powdered calcium hydride under a reduced pressure. Dibutyltin dilaurate (DBTDL) catalyst purchased from Aldrich was used as received. The disappearance of OH and NCO groups and the appearance of urethane groups were monitored by IR spectroscopy.

### 2.2. Synthesis of Multiblock Thermoplastic Polyurethane Urea

**Scheme 1:**
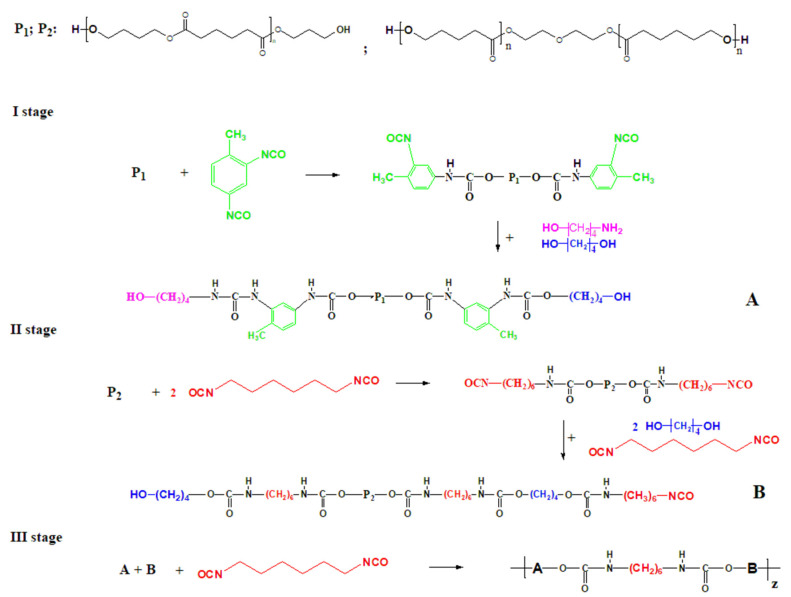
General scheme of synthesis of macrodiol A (I stage), macroisocyanate B (II stage) and studied TPUUs (III stage).

The synthesis of multiblock copolymers was carried out by the three-reactor method developed earlier by us [15]. At the first stage (reactor 1) of TPUU synthesis, a macrodiol from polydiol (PBA or PCL) and TDI in the presence of two chain extenders (BD and EA) was prepared. In the second stage (reactor 2), a macrodiol from polydiol (PBA or PCL), HMDI, and chain extender (BD) were synthesized. Then, the reaction products from the two reactors were mixed and linked by HMDI added in a stoichiometric ratio [NCO]/[OH] = 1. In the result, four polymers have been synthesized, where PCL and PBA are linked in different combinations with bulky aromatic TDI and more flexible and elongated HMDI (Table 1). Upon reaching the degree of conversion of NCO groups ≈98%, the reaction mass was poured into a flat Teflon container and dried at 40 °C during the day until constant weight. The control of the full course of the reaction was carried out by infrared spectra on the complete disappearance of absorption bands of isocyanate (νNCO = 2271 cm^−1^) and hydroxyl groups (νOH = 3620 cm^−1^). Molecular characteristics of the TPUUs were determined by gel permeation chromatography (GPC) in tetrahydrofuran using a Waters GPCV 2000 chromatograph equipped with refractometric and viscosimetric detectors. The number and weight average molecular weight of all four polymers were the following: Mn = 40,000 Da, Mw = 80,000 Da, Mw/Mn = 2. The scheme of synthesis of TPUM is presented in Scheme 1.

The molar ratio of the reaction was determined from which HS content was estimated. The hard segment (HS) was defined as:

$$\mathrm{HS}=\frac{m\left(\mathrm{TDI}+\mathrm{HMDI}\right)+m\left(\mathrm{AE}+\mathrm{BD}\right)}{m\left(\mathrm{TDI}+\mathrm{HMDI}\right)+m\left(\mathrm{AE}+\mathrm{BD}\right)+\mathrm{mPolyols}}.$$

Table 1 shows the composition and ratio of components in the synthesized TPUUs.

### 2.3. Experimental Techniques

The structural investigation was performed at the BM26 beamline of the European Synchrotron Radiation Facility (ESRF) in Grenoble, France. The beamline is equipped with Pilatus 1M (SAXS, s-range 0.002–0.04 Å^−1^) and Pilatus 300k (WAXS, s-range 0.08–0.5 Å^−1^) detectors. The experiments used X-ray photons with a wavelength of 1.04 Å, and the spot size of the beam on the sample was 0.65 × 0.65 mm. The central part of the dogbone-like test bars for analysis had dimensions of 22 × 6 × 0.7 mm. The in situ strain experiments were carried out in a Linkam TST350 tensile stage with a maximum force of 20N and strain rate of 1 mm/min. The strain rate was decreased compared to tensile tests to minimize the variation of deformation during SAXS/WAXS exposure (12 s). Shape recovery was measured during heating of stretched film at a heating rate 5 °C/min to a temperature above the melting point of the soft block e.g., 80 °C, and rapid cooling to room temperature. The two-dimensional diffraction patterns were analyzed using a program package developed in Igor Pro Program package (Wavemetrics Ltd., Portland, OR, USA) [44,45,46].

The ex situ tensile tests were performed on a Zwick TC-FR010TH Material Testing Machine at 50 mm/min stretching rate using the same samples as for SAXS/SAXS strain experiments. This test was performed according to the ASTM-D638 Type IV.

## 3. Results and Discussions

**Figure 1:**
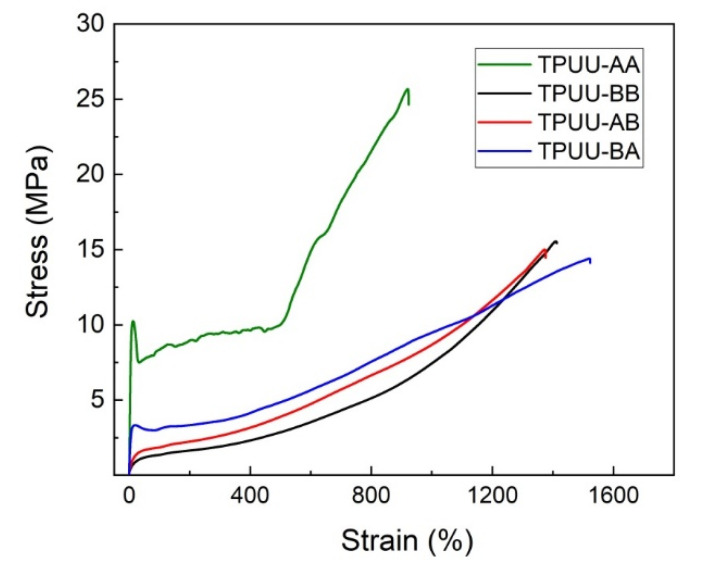
Stress–strain curves of the studied TPUUs.

The stress–strain curves of TPUU depending on the composition of the soft block are shown in Figure 1. It can be seen that TPUU-BB with PCL as a soft block demonstrates the stress–strain curve of a typical elastomer with low Young’s modulus (E) and the highest elongation at break (ε_r_) (cf. black curve in Figure 1). The elasticity in TPUU-BB is provided by the connection of PCL with a more flexible linear urethane fragment. The mechanical characteristics of TPUUs are summarized in Table 1. TPUU-AA and TPUU-BA containing the PBA block linked with HMDI demonstrate the mechanical behavior typical of stiff thermoplastics: high Young’s modulus and plastic flow with the formation of a neck in the strain range of 50–400% (green and blue curves in Figure 1). The stiffness of the materials stems from a limited mobility of PBA chains bounded with bulky rigid TDI-based urethane (Figure 1). This clearly indicates that the type of polydiol is important in the design of SME polymers. The mechanical characteristics are summarized in Table 2.

The variation of mechanical properties is related to the specific morphology of the films with different bonding of urethane of polydiol. In this paper, the reorganization of the morphology was studied in situ by SAXS/WAXS analysis using a brilliant synchrotron source.

**Figure 2:**
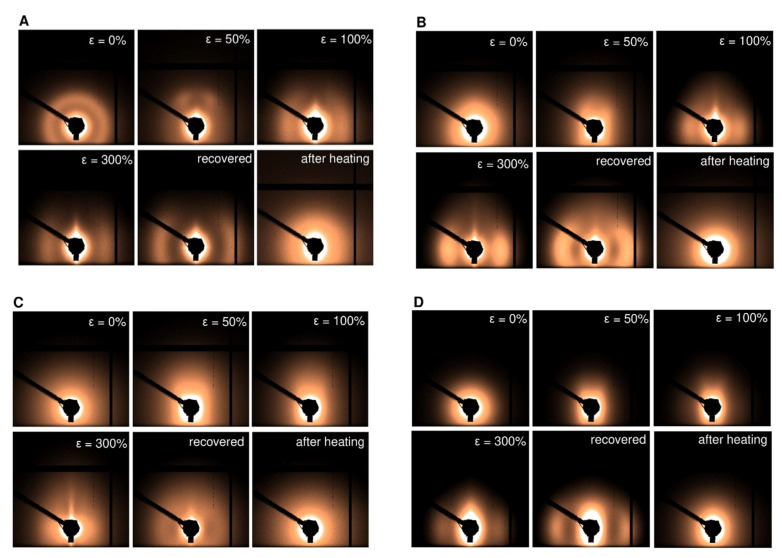
Two-dimensional (2D) SAXS patterns of the studied films: (**A**) TPUU-AA; (**B**) TPUU-BB; (**C**) TPUU-AB; (**D**) TPUU-BA, measured during strain, recovered after release of stress, and after heating to 60 °C. Drawing direction is horizontal.

**Figure 3:**
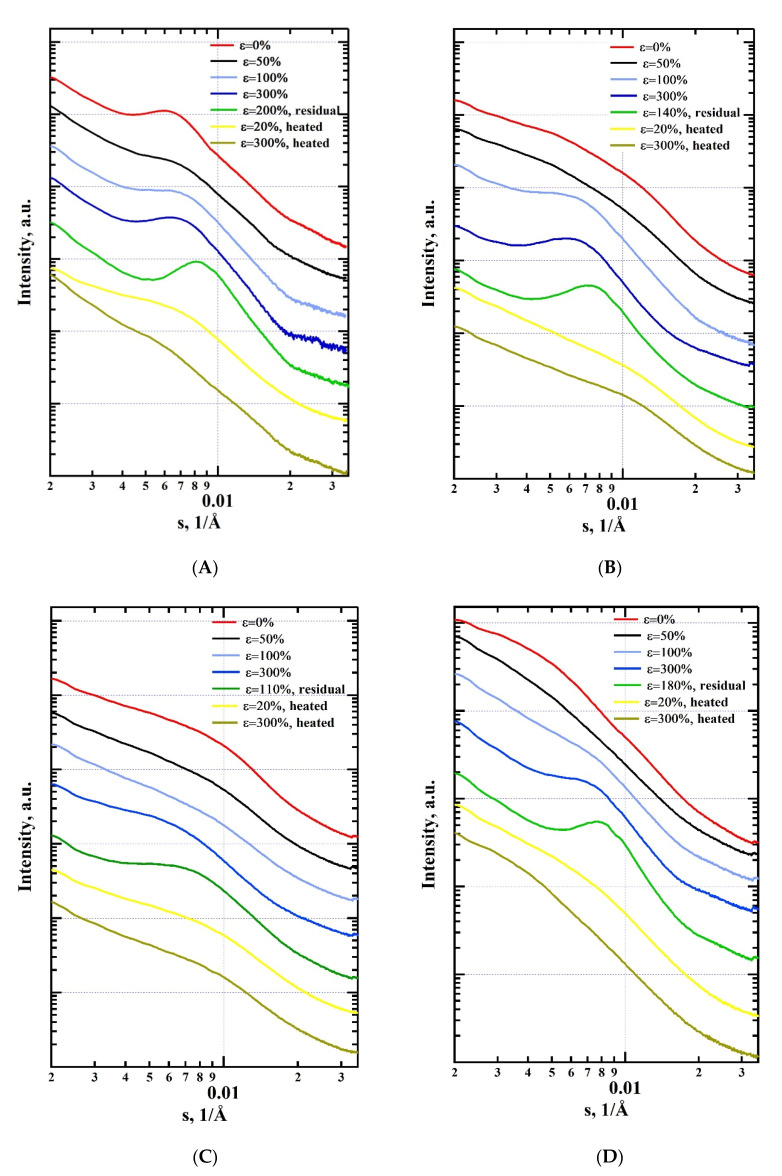
One-dimensional (1D)-reduced SAXS curves of the studied films: (**A**) TPUU-AA; (**B**) TPUU-BB; (**C**) TPUU-AB; (**D**) TPUU-BA, measured during deformation, recovered after release of stress, and after heating to 60 °C.

**Figure 4:**
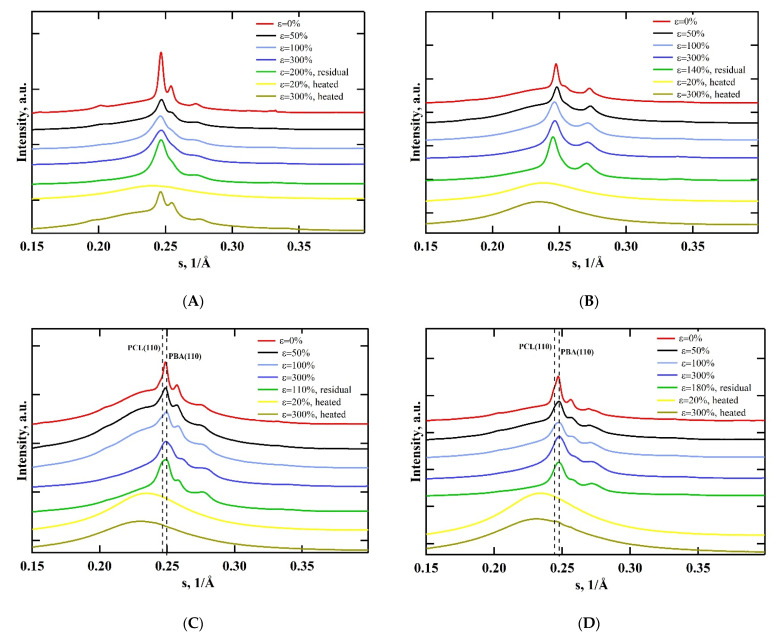
One-dimensional (1D)-reduced WAXS curves of the studied films: (**A**) TPUU-AA; (**B**) TPUU-BB; (**C**) TPUU-AB; (**D**) TPUU-BA, measured during deformation, recovered after release of stress, and after heating to 60 °C. Vertical dashed lines point on positions of the (110) peaks of PBA and PCL.

**Figure 5:**
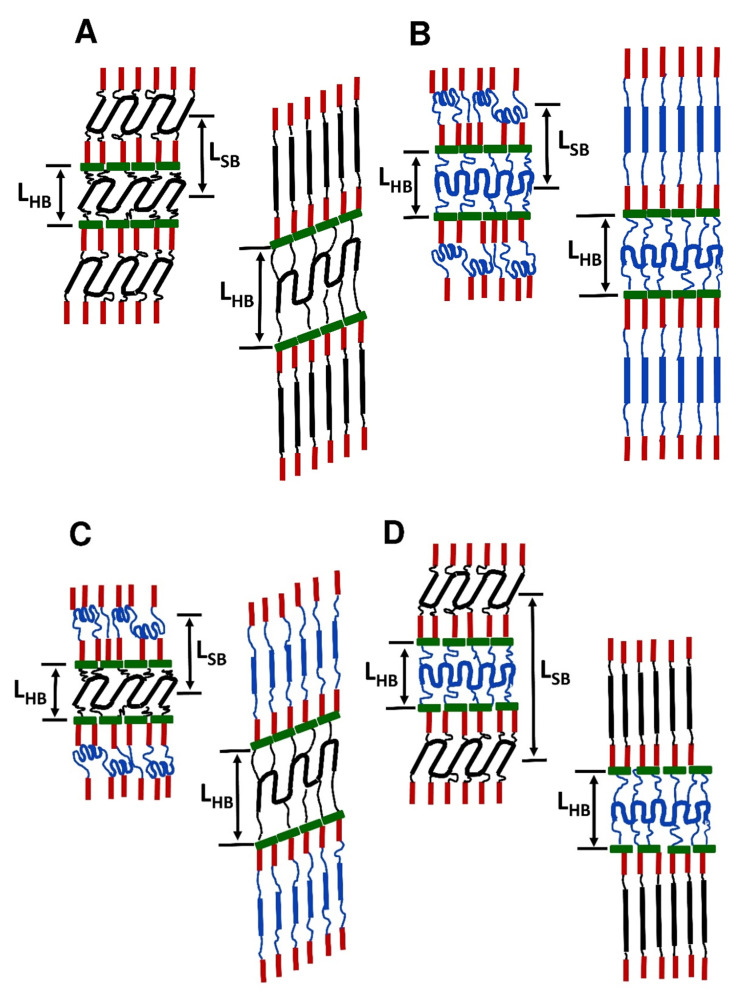
Schematics of morphology of the TDI (green), HMDI (red), PBA (black), and PCL (blue) regions in isotropic (left) and stretched to ε = 300% (right) films: (**A**) TPUU-AA; (**B**) TPUU-BB; (**C**) TPUU-AB; (**D**) TPUU-BA. Stretching direction is vertical.

Figure 2 shows changes in the film organization during stretching. Isotropic sample TPUU-AA reveals a ring characteristic of PBA crystalline lamellar ordering with a long period of L_SB_ = 14.7 nm (Figure 2A). In addition, on the 1D-reduced curve, a broad peak with a maximum at 9.7 nm can be identified (Figure 3A, black curve). This peak cannot be attributed to the second order of the long period, and its origin can be understood using the in situ film deformation experiment. Under small reversible strains, the isotropic ring transforms into a four-spot pattern, indicating reorientation of the PBA lamellae along the drawing direction [47]. An increase of half-width of the crystalline peaks in the WAXS region indicates the fragmentation of large PBA lamellae under stress (Figure 4A, blue curve). The d-spacing of the second peak increases to 10.4 nm, and it organizes in broad “clouds” positioned along the direction of stretching (Figure 3A, blue curve). At further strains, the four-spot lamellar peak disappears due to the lack of phase contrast during plastic flowing of the material in the neck. Meantime, the equatorial orientation of the second peak becomes more pronounced, and its position gradually increases to 12.9 nm for ε = 300% (Figure 3A, dark blue curve). After release of stress, the film of TPUU-AA shows high residual deformation—200% in the neck. The intensity of the second peak increases because of enhanced phase contrast and the spacing decreases to L_HB_ = 11.6 nm (Figure 3A, green curve). Interestingly, this peak is preserved even after heating to above the melting point of the PBA crystal phase (Figure 3A and Figure 4A, yellow curves). Consequently, the peak can be assigned to the phase-separated morphology of soft and hard segments rather than to the stacking of PBA crystals [15]. We expect that this spacing reflects the distance between the rigid TDI domains that surround the PBA block (see Figure 5A, left). The PBA chains bonded to TDI crystallize under hard geometric constraints [48,49]. The presence of stressed tie chains in such domains prevents the constrained PBA lamellae from unfolding during stretching with small elastic deformation of the amorphous phase (Figure 5A, right). In contrast, the PBA lamellae surrounded by relatively flexible linear urethane fragments are formed in soft confinement. During application of strain, these regions behave as typical semi-crystalline flexible-chain polymers with plastic flow and the formation of fibrillar crystals. The difference in crystallization conditions of PBA connected to aromatic and linear urethanes can illustrate the appearance of SAXS maximum at 20.4 nm during the deformation of amorphous TPUU-AA after heating to 80 °C (Table 3), indicating crystallization of the HMDI-surrounded soft blocks before the TDI-surrounded ones. Two populations of the PBA crystals generate two melting peaks in the DSC curve of the fresh TPUU-AA sample [14].

The replacement of well-crystallizable PBA soft block by PCL with a moderate crystallization rate at room temperature changes the morphology and corresponding mechanical characteristics of TPUU (Figure 2B). In the isotropic film, the lamellar SB peak is broader and shifted to smaller angles (Figure 2B and Figure 3B, red curve). During deformations up to 50%, one can see an increase of both SB and HB periodicities (Figure 3B, black curve, Table 3). The intensity of the SB peak is concentrated in the direction normal to drawing (Figure 2B). On further stretching, the SB maximum from folded lamellae disappears without plastic flow of PCL chains (Figure 2B and Figure 3B, blue curve). At even higher deformations, WAXS reveals growing crystalline peaks corresponding to the stress-induced formation of fibrillar PCL crystals (Figure 4B, blue curve). For ε = 300%, one observes a certain decrease of phase-separated L_HB_ distance probably caused by “sacrifice-and-relief” mechanism described by Stribeck for MDI-based polyurethanes [39].

The same effect of decrease of L_HB_ was detected during stretching of the amorphous film preliminarily heated above the melting point of PCL (Table 3). The peak due to phase separation is less pronounced probably because of the better miscibility of PCL with linear urethanes (Figure 3B, yellow curve). Interestingly, stretching of the initially amorphous film does not show any stress-induced crystallization (Figure 3B, brown curve). Generally, TPUU-BB with a PCL soft block possesses less regular lamellar organization. Rubber-like stress–strain curves and a higher increase of L_SB_ and L_HB_ indicate that both TDI-bounded and HMDI-bounded PCL chains do not form regularly folded lamellae.

In the case of TPUUs with both PBA and PCL soft blocks, an important role is played by the position of a particular polydiol in the multiblock chain. Isotropic sample TPUU-AB reveals the presence of a mainly crystalline phase of TDI-bounded PBA (Figure 4C, red curve). We suppose that PCL blocks surrounded by semi-flexible linear urethanes still have a low crystallization rate, resulting in the absence of correlation between PCL and PBA lamellae (Table 3). During deformation, the crystal fraction of PCL increases due to stress-induced crystallization and the weak SAXS maximum at L_SB_ = 18.4 nm is visible up to ε = 100% (Figure 3C and Figure 4C blue curves). PBA crystals surrounded by hard domains of TDI show a relatively small increase of L_HB_ on deformation and absence of the peak orientation in the drawing direction (Figure 2C and Figure 3C). After the release of stress, the orientation of the phase-separated morphology almost completely disappears due to the high flexibility of the PCL blocks (Figure 3C, green curve). This sample demonstrates mechanical behavior that is intermediate between rubber-like and thermoplastic without necking but with orientational hardening (Figure 1, red curve and Figure 5C).

In contrast, WAXS patterns of TPUU-BA with HMDI-bounded PBA chains reveal a high degree of crystallinity in the isotropic state with an approximately equal content of PBA and PCL crystals (Figure 4D, red curve). Thus, PCL chains constrained by a neighbor TDI hard domain show a better tendency to crystallize at room temperature than in the more mobile HMDI domains (Figure 5D). However, regular lamellae of HMDI-surrounded PBA blocks give high phase contrast, resulting in the enhanced SAXS maximum with increased distance L_SB_ = 20.6 nm (Figure 3D, red curve). This peak transforms to a four-spot pattern at ε = 50% but further disappears due to the lack of phase contrast between HB and extended-chain PBA crystals (Figure 2D and Figure 3D, blue curve). Interestingly, PCL chains in the TDI domain do not reveal significant deformation and stress-induced crystallization (Figure 4D, dark blue curve). In contrast to TPUU-AA, the HB peak of TPUU-BA shows very good azimuthal orientation in the drawing direction at high strain (Figure 2A,D). This can be attributed to bigger chain tilt in constrained PBA lamellae compared to the lamellae of PCL (Figure 5A,D). After heating above the melting point of PBA and PCL crystals, HMDI-surrounded PBA chains rapidly crystallize with the reappearance of maximum at 24.6 nm during the stretching of amorphous film (Figure 3D, yellow curve). This confirms that the crystallization of HMDI-surrounded PBA chains occurs faster than that of TDI-surrounded PBA with the formation of more regularly stacked lamellae. The presence of HMDI-surrounded PBA in TPUU provides relatively high residual deformation after the release of stress and a slow relaxation of strain that is important for the design of shape memory materials.

## 4. Conclusions

In conclusion, the variation of chemical nature of polydiol and adjusted diisocyanates allows tuning the mechanical properties of resulting TPUUs from soft elastomers to rigid thermoplastics. Synchrotron SAXS/WAXS studies of a series of TPUUs reveal a complex change in morphology during deformation related to the superposition of phase separation of soft and hard blocks and crystallization of the polydiols. It was shown that TDI-bounded polydiols are constrained in rigid domains which, on the one hand, decrease the crystallization rate and regularity of lamellae but, on the other hand, preserve crystals from plastic flow during strain. In this case, constrained crystals of soft block play the role of an additional physical network imparting higher stiffness to the material. In the meantime, HMDI-bounded polydiols reveal higher crystallinity and faster crystallization from the melt. During deformation, these crystalline domains behave as typical semi-crystalline flexible-chain polymers. The TPUUs with HMDI-bounded PBA show plastic flow with the formation of extended-chain crystals, while HMDI-bounded PCL fragments reveal stress-induced crystallization. Thus, HMDI-rich domains are responsible for the elastic characteristics of the material: elongation at break, residual deformation, etc. Consequently, rigid aromatic TDI or semi-rigid linear HMDI form a different interface for two populations of crystallites of polydiols. A significant difference in crystallization kinetics at room temperature between PBA and PCL provides an additional tool for fine-tuning the thermoplastic properties and shape memory behavior.

In situ investigation of the structure and morphology of TPUU at different external stimuli allows finding the relationship between the structure and deformational properties that helps optimizing the composition of the soft block for desired mechanical characteristics.

## Figures and Tables

**Table 1:** Chemical composition of studied TPUUs.

Polymer	Polymer Composition		Mass Fraction of Reagents, %			
	P1	P2	Polyol	Diisocyanate	Chain Extender	SS/HS
TPUU-AA	PBA	PBA	69	23	8	2.2
TPUU-BB	PCL	PCL	69	23	8	2.2
TPUU-AB	PBA	PCL	69	23	8	2.2
TPUU-BA	PCL	PBA	68	23	8	2.2

**Table 2:** Mechanical characteristics of TPUUs.

Polymer	ε_r_, %	σ_r_, MPa	E, MPa
TPUU-AA	807 ± 78	22 ± 3	199 ± 4
TPUU-BB	1445 ± 27	15 ± 1	7 ± 1
TPUU-AB	1445 ± 52	16 ± 1	11 ± 1
TPUU-BA	1340 ± 84	13 ± 2	59 ± 2

**Table 3:** Structural parameters of samples at different strain.

Polymer	ε, %	L_HB_, nm	L_SB_, nm
TPUU-AA	0	9.7	14.6
	50	10.8	14.0
	100	12.8	-
	300	12.9	-
	200, restored	11.6	-
	20, after heating	9.7	-
	300, after heating	9.4	20.4
TPUU-BB	0	9.6	15.6
	50	10.3	17.8
	100	14.7	-
	300	14.3	-
	140, restored	12.5	-
	20, after heating	9.1	-
	300, after heating	8.6	-
TPUU-AB	0	10.8	-
	50	11.2	16.3
	100	12.4	18.4
	300	14.8	-
	110, restored	12.3	-
	20, after heating	10.1	-
	300, after heating	9.9	-
PTUU-BA	0	10.0	20.6
	50	11.7	24.8
	100	13.6	-
	300	13.0	-
	180, restored	11.4	-
	20, after heating	11.1	-
	300, after heating	13.8	24.6

## Data Availability

The data presented in this study are available in article.
